# Potential Genetic Contributions of the Central Nervous System to a Predisposition to Elite Athletic Traits: State-of-the-Art and Future Perspectives

**DOI:** 10.3390/genes12030371

**Published:** 2021-03-05

**Authors:** Hiroya Kitazawa, Kazuya Hasegawa, Daichi Aruga, Masashi Tanaka

**Affiliations:** 1 Department of Physical Therapy, Health Science University, 7187 Kodachi, Fujikawaguchiko-machi, Minamitsuru-gun, Yamanashi 401-0380, Japan; blackf12c@icloud.com (H.K.); a830daichi@icloud.com (D.A.); 2 Faculty of Nutritional Sciences, Morioka University, 808 Sunakomi, Takizawa City, Iwate 020-0694, Japan; hasegawa117h@yahoo.co.jp

**Keywords:** central nervous system, dopaminergic system, elite athletic traits, genetic factors, serotonergic system

## Abstract

Recent remarkable advances in genetic technologies have allowed for the identification of genetic factors potentially related to a predisposition to elite athletic performance. Most of these genetic variants seem to be implicated in musculoskeletal and cardiopulmonary functions. Conversely, it remains unclear whether functions of the central nervous system (CNS) genetically contribute to elite athletic traits, although the CNS plays critical roles in exercise performance. Accumulating evidence has highlighted the emerging implications of CNS-related genes in the modulation of brain activities, including mental performance and motor-related traits, thereby potentially contributing to high levels of exercise performance. In this review, recent advances are summarized, and future research directions are discussed in regard to CNS-related genes with potential roles in a predisposition to elite athletic traits.

## 1. Introduction

Potential factors affecting athletic performance have become a topic of great interest. Accumulating evidence shows that both genetic and environmental factors are involved in athletic status [1,2], as genetic variants can modify an individual’s response to environmental influences [3]. Recent remarkable advances in genetic technologies have allowed for a deeper understanding of the relationships among genetic factors, inherent exercise capacity (EC), and athletic performance. A previous genetic study of sports-related traits of British female twins reported that approximately 66% of elite athletic performance is heritable [1]. Furthermore, an array of genetic variants, including single nucleotide polymorphisms (SNPs) of genes involved in skeletal muscle structure and function (e.g., α-actinin-3 (*ACTN3*)), blood pressure control (e.g., angiotensin-converting enzyme (*ACE*)), and energy metabolism (e.g., peroxisome proliferator-activated receptor γ (*PPARG*)), have been increasingly identified as potential markers of inherent athletic performance [2,4,5]. These findings provide insights into the genetic basis that partially accounts for the differences in inherent EC among individuals.

In addition to peripheral factors such as musculoskeletal and cardiopulmonary functions, the central nervous system (CNS) plays a pivotal role in athletic performance, as the brain controls the initiation and processing of physical activity [6], motor skill acquisition [7], regulation of physical activity [8], and psychological traits, including motivation to exercise, anxiety, and stress resilience [4,8]. In light of the relationship between the brain and physical activities, extensive studies on the effects of exercise on brain functions have reported that exercise is beneficial to cognitive function, potentially through multifactorial pathways that include improvement of cardiovascular conditions [9], adult neurogenesis [10,11], and modifications of neurotransmission by muscle-derived bioactive molecules [12,13,14]. In addition, rodent-based studies have found that differences in the inherent activities of distinct brain regions, such as the reward circuitry and cerebellum, are closely related to those in inherent voluntary participation in exercise [8,15,16]. Accordingly, it has been suggested that a crosstalk may exist between the brain and physical activities that could be influenced by genetic factors. However, the contribution of brain activity to inherent elite athletic status remains unclear.

Elucidation of the potential link between inherent brain activity and EC would be helpful to develop novel personalized CNS-targeted strategies to improve exercise performance. Although studies are limited, it is increasingly evident that inherent brain activity is closely related to inherent EC. Here recent findings of the potential effects of inherent brain activity on inherent exercise potentials are reviewed and future research directions on the development of interventional strategies targeting the CNS to improve exercise performance are discussed.

## 2. Brain Areas and Functions Related to a Predisposition to High EC: Findings of Rodent-Based Studies

Based on the possibility that inherent (untrained) running capacity reflects inherent exercise-related traits, lines of rodents with higher or lower EC were established by selective breeding [6,17]. An exercise-testing protocol, rather than genetic selection, was created to select rodents with intrinsically higher and lower EC [18,19,20]. Previous biochemical studies with the use of these rodents have provided significant insights into the relationship between differences in inherent brain activities and inherent EC [6,18,19,20].

### 2.1. Emerging Roles of Pain Analgesia System

To identify patterns of brain activation involved in inherent EC, an early study examined the brains of genetically modified rats with higher vs. lower EC [6]. The authors analyzed the mRNA levels of Fos proto-oncogene, AP-1 transcription factor subunit (*c-Fos*), a transcription factor induced by dopaminergic signaling and a marker of neuronal activation [6,21], as an indicator of brain activity [6]. The results showed that the caudate putamen (CPu), primary motor cortex, and cingulate cortex, which are involved in motor control and motivation, were activated in response to exercise in both lines, but there was no significant difference in the levels of brain activity [6].

Interestingly, activities of the periaqueductal gray, nucleus raphe magnus, and locus coeruleus were higher in the brains of rats with higher EC as compared to those with lower capacity after exercise [6]. The periaqueductal gray projects to the serotonergic neurons of the nucleus raphe magnus and then to the brain stem or spinal cord, thereby leading to descending pain modulation and pain relief [22]. The locus coeruleus constitutes the descending noradrenergic pathway, which is involved in pain analgesia [23]. Accordingly, the pain analgesia system of rats with higher EC is efficiently activated to reduce the sensation of pain and elevates the threshold of pain during exercise, which could contribute to inherently higher exercise performance [6]. In this respect, the pain analgesia system presents an interesting target of elite athletic performance, although further investigations are needed to identify relevant genes and/or SNPs.

### 2.2. Differential Roles of the Dopaminergic and Serotonergic Systems

The CNS plays significant roles in exercise and fatigue, and functional interactions between the dopaminergic and serotonergic systems affect physical performance [18,24]. Dopaminergic neurons from the substantia nigra pars compacta project to the CPu and form the nigrostriatal pathway, while those from the ventral tegmental area send input to the nucleus accumbens (NAc) and constitute the mesolimbic pathway [18]. These areas have been implicated in motor control and motivation for exercise [18]. When released, dopamine (DA) binds to D1-like (D1 and D2) and D2-like (D2, D3, and D4) receptors, thereby triggering physiological responses [24,25]. Reportedly, pharmacological/nutritional intervention targeting the dopaminergic system can improve the physical performance of both rodents and humans [24].

The serotonergic system, in which serotonin (5-hydroxytryptamine (5-HT)) functions as a neurotransmitter, is formed by neurons originating in the raphe nuclei that projects to many structures of the CNS, including the CPu and NAc [18]. In contrast to the dopaminergic system, the serotonergic system is involved in lethargy and loss of motivation [18,24]. A previous study suggested that differences in inherent monoaminergic activities of the CNS contribute to differences in inherent EC [18].

To address this issue, a previous study investigated whether differences exist in the dopaminergic and serotonergic activities of the CPu and NAc of rats with and without intrinsically high EC based on an exercise-testing protocol [18]. The results revealed that the ratio of the DA metabolite 3,4-dihydroxyphenylacetic acid (DOPAC) to DA, an index of DA turnover, was elevated in the CPu of both rat groups in response to exercise, although there was no significant difference between the groups and there was no significant change in the DOPAC/DA ratio of the NAc after exercise [18]. In light of these findings based on the DOPAC/DA ratio, the intrinsic responsiveness of the dopaminergic system in the CPu and NAc to exercise appears to be unrelated to intrinsic EC.

In the case of the serotonergic system, the ratio of 5-hydroxyindoleacetic acid (5-HIAA) to 5-HT in both the CPu and NAc was elevated in response to exercise in only rats with higher EC [18], which suggests that the intrinsic responsiveness of the serotonergic system in the CPu and NAc to exercise is higher in rats with intrinsically higher EC. However, this finding was unexpected due to the potential negative effects of the serotonergic system on physical performance. The authors speculated that rats with higher EC can tolerate serotonergic activity and defer fatigue, thereby achieving high exercise performance [18]. Alternatively, elevated activities of the serotonergic system may contribute to exercise performance in a matter-of-fact manner. Moreover, stimulating the serotonergic system may be involved in improving the balance between sympathetic and parasympathetic nervous systems for high exercise performance. As another possibility, the serotonergic system has beneficial, but unidentified, roles in attainment of high levels of exercise performance. However, future studies are required to elucidate the potential roles of the serotonergic system in enhancement of exercise performance.

### 2.3. Emerging Roles of Neuronal Circuits Related to Neuroplasticity

Neuroplasticity is a process of neuronal circuit modification by altering the structure and function of the synapses in response to behavioral changes [26]. Accordingly, it would be beneficial to efficiently modulate neuroplasticity in response to physical training in order to attain a high level of exercise performance. A previous study investigated the effects of training (running on a motor-driven treadmill for six weeks) on time of exercise and dopaminergic and serotonergic activities in the CPu of rats with and without intrinsically high EC that were selected with an exercise-testing protocol [19]. The results showed that physical training improved the time of exercise of rats with higher EC as compared to that of sedentary control rats, although there was no increase in the DOPAC/DA ratio in the CPu, but rather a decrease, and there was no significant effect on the 5-HIAA/5-HT ratio [19]. Furthermore, physical training had no significant effect on the expression levels of genes related to the dopaminergic system encoding the D1 receptor (*Drd1*), D2 receptor (*Drd2*), dopamine transporter (*Dat*), and glial cell line-derived neurotrophic factor (*Gdnf*) in the CPu [19]. Accordingly, the intrinsic activities of the dopaminergic and serotonergic systems in the CPu are not significantly related to neuroplasticity elicited by physical training in rats with higher EC.

Conversely, in rats with lower EC, physical training resulted in an increase in the DOPAC/DA ratio, but not the 5-HIAA/5-HT ratio, and changes in the expression levels of genes related to the dopaminergic system in conjunction with an improvement in the time of exercise [19]. These results suggest that the responsiveness of dopaminergic neuroplasticity to physical training is intrinsically higher in rats with intrinsically lower EC, which further support the findings that exercise performance was more enhanced after physical training in rats with lower EC as compared to those with higher EC [19]. These findings suggest that rats with lower EC have more efficient neural adaptation and trainability [19], as physical training could compensate for the potential unfavorable genetic predisposition and confer more improved exercise performance, which would lead to lower inter-individual differences in physical performance [19].

Recent exercise and molecular studies identified various genes involved in exercise-related neuroplasticity of isolated rodent brains, such as the N-methyl-d-aspartate receptor subunit genes (*Grin1* and *Grin2b*) [27], hypoxia-induced factor 1 α (*Hif1a*) [28], and cyclic adenosine monophosphate response element-binding protein (*CREB*) [29]. The N-methyl-d-aspartate receptor plays a pivotal role in synaptic development and plasticity [27,30]. HIF-1α is a transcription factor that stimulates expression of an array of genes involved in various physiological functions, including metabolism, angiogenesis, and synaptogenesis [28]. CREB is also a transcription factor that is closely implicated in neuronal differentiation and synaptic plasticity [29]. The potential roles and mechanistic details underlying the inherent brain activities of these factors in exercise performance in humans have not been fully elucidated. Additional studies focusing on these genes would be helpful to elucidate the molecular basis of the relationship between inherent neuroplasticity and inherent EC.

### 2.4. Potential Roles of Thermoregulation-Related Factors

Thermoregulation efficiency also has a significant effect on physical performance. As muscle contraction increases heat production, good overall health and a high level of physical performance are necessary to maintain core body temperature in response to exercise [20,31]. A recent study investigated whether differences in intrinsic thermoregulatory capacity are related to difference in intrinsic EC using rats with and without intrinsic high EC that were selected with an exercise-testing protocol [20]. The authors found that an increase in abdominal temperature in response to exercise was more efficiently suppressed in rats with higher EC than those with lower EC [20]. The results further revealed that there was no significant difference between groups in the DOPAC/DA ratio in the preoptic area of the hypothalamus [20], which is the primary integrative brain area responsible for thermoregulation [32]. Conversely, concentrations of both DOPAC and DA were more increased in response to exercise in rats with higher EC as compared to those with lower EC [20]. Accordingly, the responsiveness of DA synthesis and metabolism in the preoptic area to exercise would be intrinsically higher in rats with higher EC, which might be implicated in the higher thermoregulation efficiency [20]. Oxygen consumption throughout exercise was lower, but the highest values of oxygen consumption and mechanical efficiency was higher in rats with higher EC, suggesting that heat production during exercise is intrinsically lower in rats with higher EC, which contributes to higher thermoregulation efficiency [20]. Although genetic factors related to efficient thermoregulation of high EC remain unclear, the dopaminergic system in the preoptic area presents a potential target for future studies.

### 2.5. A Summary of Findings of Rodent-Based Studies

As summarized in Table 1, rodent-based studies have identified several functions of the CNS that are potentially related to a predisposition to high EC. Genes involved in high EC have not been identified in rodent-based studies. However, biochemical and molecular biological analyses of brains isolated from rodents—a limitation of human studies—have uncovered brain functions, including pain–analgesia, serotonergic, dopaminergic, and thermoregulatory systems (Table 1), which contribute to inherently high EC.

Intrinsically high EC would provide a survival benefit to rodents, but the notion of athletic competition cannot be applied to rodents. Therefore, it is possible that CNS functions, identified in rodent-based studies, predispose the animals to high EC, which would not include human characteristics, such as emotional aspects. In this respect, characteristics common to these species could support the extrapolation of animal findings to humans, consistent with the report that the ergogenic effects of the dopaminergic system were present in both rodents and humans [24]. Findings of rodent-based studies are not necessarily translatable to humans, and attention should be paid to species differences. Nevertheless, genetic association studies in humans have increasingly identified CNS-related genetic factors, which are also related to high EC in rodents (Table 1). In humans, some of these factors might have additional roles in conferring elite athletic traits through human traits, such as mental, emotional, or mental and emotional control. These novel aspects are reviewed in the next section.

## 3. CNS-Related Genes That Potentially Contribute to a Predisposition to Elite Athletic Traits: Findings of Human Studies

In humans, more than 200 genetic variants are reportedly related to physical performance, including at least 155 that have been linked to elite athletic status [33,34]. These findings indicate the inheritability of elite athletic traits. Although most of these variants are likely involved in peripheral functions, the genetic contribution of the CNS to high levels of athletic performance has not been fully elucidated. This might be attributable to polygenic effects brought about by a large number of genes with small impacts [33]. Because of the complexity of motor coordination, it is difficult to examine the effects of genetic polymorphisms on athletic skill performance [35]. However, it is increasingly evident that several genes are potential regulators of elite athletic traits through functions of the CNS.

### 3.1. Genes Related to Motor Skills, Motor Learning, Motor Performance, and Sleep

The acquisition, attainment, and coordination of high levels of motor skills are closely related to elite athletic traits. In this context, to identify the brain activity-related genetic variants associated with elite athletic status, it could be effective to analyze elite players in sports that involve the use of equipment or apparatus manipulation.

Australian rules football (ARF) is a multi-dimensional team sport in which players need high levels of skill performance, such as kicking and handballing skills, and to constantly modify behaviors in response to the changing situation of the game [35,36]. In light of these aspects of ARF, a recent study investigated whether several genetic polymorphisms of interest in sub-elite ARF players were associated with ARF-specific skill sets (kicking and handballing) and game performance (direct game involvement per minute) [35]. The results revealed that the brain-derived neurotropic factor (*BDNF*) A/G (rs6265) genotype was significantly associated with game kicking performance [35], and the *DRD2* A/A (rs1076560) and β 1 adrenergic receptor (*ADRB1*) C/C (rs1801253) genotypes were significantly associated with handballing performance [35]. In non-athletes, the *BDNF* genotype has been suggested to be involved in modulating the interhemispheric transfer of a procedural motor skill [37] and the *DRD2* genotype has been related to motor learning and performance [38]. Accordingly, the *BDNF* and *DRD2* genotypes might be novel CNS-related genetic factors involved in a predisposition to brain activities for elite levels of skill performance.

Conversely, the *ADRB1* genotype has been associated with endurance performance [5,35], but the contribution to the relationships of CNS functions and elite athletic performance remains unclear. In this respect, based on genetic and experimental approaches, a recent study has uncovered novel roles of ADRB1 that might be involved in a predisposition to elite athletic traits; ADRB1 has a significant role in the regulation of sleep and wakefulness [39]. The authors showed that ADRB1 was highly expressed in the dorsal pons, a part of the brain stem involved in sleep regulation, and the *ADRB1* mutation Ala187Val (C/T (rs776439595)) activated relevant neurons, thereby leading to a natural short (but sufficient) sleep trait in both humans and mice [39]. The quantity and quality of sleep are closely implicated in physical performance, and athletes are at a high risk of sleep difficulties, including insufficient duration and poor quality. Thus, the promotion of sleep health could significantly improve the performance of elite athletes [40]. Although relative contributions of *ADRB1* C/C (rs1801253) and C/T (rs776439595) on sleep regulation warrant further investigation, the *ADRB1* genotype may be a novel genetic contributor to elite athletic traits through its effects on sleep health.

Furthermore, individuals with a short sleep trait are healthy and energetic with a high pain threshold [41]. Although mechanistic details underlying the relationship between sleep trait and pain relief remain unclear, the potential contribution of the pain analgesia system to a predisposition to higher EC has been reported in rodents [6]. These findings suggest potential links of sleep, pain, and elite athletic status. Therefore, genes involved in the regulation of sleep and/or pain present attractive targets for future studies to identify novel CNS-related genes related to a predisposition to elite athletic traits.

Analysis of sub-elite ARF players further identified three genotypes associated with match performance, as measured by direct game involvement per minute: *DRD2* A/A (rs1076560), *ADRB1* C/C (rs1801253), and peroxisome proliferator-activated receptor γ, coactivator 1 α (*PPARGC1A*) A/G (rs8192678) [35]. The *PPARGC1A* rs8192678 genotype may have a role in the efficiency of aerobic metabolism [42] and has been suggested to be associated with endurance performance [35,42].

Overall, these findings suggest that genetic factors that modulate brain activity, as well as those affecting peripheral functions, contribute to a predisposition to high levels of player performance in team sports that require skill performance and behavior modification. Therefore, these genetic factors would be helpful to develop individualized training programs to effectively improve performance by predicting the player’s response to an individualized training regime [35].

### 3.2. Genes Related to the Serotonergic System

To identify genetic variants with the potential to modulate brain activity, another study investigated potential associations of genes of interest with elite athletic traits by focusing on genetic factors commonly related to the traits of elite athletes participating in endurance, power, and combat sports [34]. The results revealed that the fifth Ewing variant (*FEV*) G/A (rs860573) genotype was associated with elite athletic status across all sport groups [34]. FEV is a transcription factor exclusively involved in the differentiation and maintenance of serotonin neurons [34,43]. Previous studies have reported that FEV plays a significant role in normal anxiety-related behaviors [34,44], and impaired serotonergic function in CNS leads to psychiatric diseases, including aggressiveness and impulsive behaviors [34,45,46]. In light of the function of FEV and the potential role of the serotonergic system in psychiatric diseases, the authors speculated that the serotonergic system has functional significance in elite athletic performance. For optimal athletic performance, calm planning, execution of trained actions, and mental quality are required. However, impairment of the serotonergic system could result in mental and/or emotional derangement, thereby negatively affecting the attainment of high performance levels [34]. Accordingly, an elevation in serotonergic activity could be helpful to achieve optimal performance in athletic competitions [34]. Since there are only slight differences in the physical traits of top-level athletes, optimal physical and mental performance is key to success in athletic competitions [34,47]. The serotonergic system has been suggested as a genetic contributor to elite athletic status [34]. However, future studies are needed to elucidate the mechanisms underlying the relationship of the *FEV* genotype, serotonergic system, and athletic performance.

In addition to the aforementioned human study [34], potential roles of the serotonergic system in intrinsically higher EC have also been reported in rodents [18]. However, when subjected to an exercise task [18], rodents do not require the level of athletic competition or mental capacity required of human competitors in sports. Therefore, the serotonergic system may have novel roles in the attainment of high performance levels, other than mental and/or emotional control. Accumulating evidence suggests that serotonin neurons are non-homogeneous in terms of anatomical, biochemical, and electrophysiological characteristics, and sub-populations of serotonin neurons form complex neuronal circuits through interconnections [46]. It is intriguing to speculate the existence of subtypes of serotonin neurons related to athletic performance, which could be attractive targets for future studies.

### 3.3. Genes Related to the Dopaminergic System

Physical performance is reportedly influenced by functional interactions between the dopaminergic and serotonergic systems [18,24]. It has also been reported that pharmacological/nutritional intervention targeting the dopaminergic system improved the physical performance of both humans and rodents [24]. However, the potential genetic contribution to a predisposition to high levels of exercise performance seems to be more evident in the serotonergic system than the dopaminergic system, which raises the issue of why dopaminergic circuits are not detected as remarkable genetic factors for a predisposition to elite athletic traits.

Reportedly, behavioral and neurophysiological responses to the amount of DA undergo an inverted U-shaped function [48,49]. Low DA levels in the brain have been implicated in fatigue and inertia, whereas high levels result in increased heart rate and symptoms of nervousness and insomnia [49]. Thus, to improve physical performance, appropriate amounts of DA should be produced and utilized for neurotransmission in the brain. In this respect, genetic variants of dopaminergic circuits, such as the SNP *DRD2/*ankyrin repeat and kinase domain containing 1 (*ANKK1*), would cause differences among individuals in the optimal DA dose of the inverted U-shaped function [48]. In addition, behavior is influenced by both endogenous and exogenous stimulation of specific neuronal circuits [48], which might also be affected by genetic variants. Hence, the combination of several genetic variants contributes to the optimal DA dose, thereby possibly making it difficult to isolate genotypes related to the dopaminergic system as a predisposition to elite athletic traits, as individual genes with only a slight effect might not be detected, even though the combination of such genes contributes to elite athletic status through attainment of optimal dopaminergic functions in the brain.

Another notable aspect of the dopaminergic system in the CNS is its roles in regulating aggressiveness. Elevated DA activity is associated with aggressive behavior, and DA signaling in the prefrontal cortex (PFC) is involved in modulating aggressiveness [50]. In light of these characteristics, a recent study examined whether genetic differences in DA levels in the brain contribute to a predisposition to elite athletic status in combat sports by focusing on the gene encoding catechol-O-methyltransferase (*COMT*), a catecholamine-catabolizing enzyme expressed throughout the brain [50].

The Val158Met (rs4680) polymorphism of the *COMT* gene reduces enzymatic activity, thereby resulting in elevated DA levels [50]. Interestingly, elite fighters in mixed martial arts were found to carry the *COMT* Val genotype (G/G), in which baseline levels of DA are decreased by high enzymatic activity as compared to non-athletes [50]. Since DA levels are elevated by increased stress [51], the authors discussed that these results were in line with the inverted U-shaped curve theory, as stress-induced DA during a match would confer the optimal DA dose to Val carriers (lower levels of baseline DA in the PFC), whereas the DA dose of Met carriers (higher levels of baseline DA in the PFC) would be outside of the optimal range [50]. Accordingly, the *COMT* Val genotype might be a novel genetic contributor to a predisposition to elite athletic traits in combat sports requiring aggressiveness under high stress conditions [50]. These findings also suggest that environmental factors, such as stress conditions, and genetic factors affect athletic performance, thereby causing difficulty in the identification of genes involved in the dopaminergic system for a predisposition to elite athletic status.

### 3.4. Transcranial Direct Current Stimulation (tDCS) and Genes Potentially Related to Responsiveness to tDCS

A non-pharmacological/non-nutritional intervention strategy, tDCS, has recently become a topic of interest as a potential novel approach to improve athletic performance through electrical stimulation of brain areas of interest [52,53,54]. It has been reported that tDCS was beneficial to the exercise performance of elite athletes as well as of untrained volunteers [52]. Conversely, it has been argued that scientific evidence is still insufficient to support the ergogenic effects of tDCS, as significant challenges must be resolved, such as stimulation intensity, sites of stimulation, and inter-individual variabilities [53]. However, recent advances in tDCS-related technologies have allowed a better understanding of the neurophysiological effects of tDCS on the brain [54].

A previous study reported that anodal tDCS delivered to the primary motor cortex improved motor learning and memory, in parallel with a reduction of the stimulated region of γ-aminobutyric acid (GABA) [55], an inhibitory neurotransmitter involved in motor learning [54,56]. Accordingly, tDCS was reported to influence exercise performance by modulating neurotransmitters in the CNS [54,55]. Although genes related to the GABAergic system have not been identified as genetic contributors to a predisposition to elite athletic status, future studies are expected to uncover genetic variants of neurotransmitters and/or receptors related to the GABAergic system. These findings further suggest that inter-individual variabilities in responsiveness to tDCS would partially be attributable to differences in genetic factors affecting brain activity, such as the types and levels of neurotransmitters and related receptors. In this context, genetic traits related to the functions of the CNS would be implicated in the potential ergogenic effects of tDCS.

### 3.5. A Summary of Findings of Human Studies

As described above, accumulating evidence highlights emerging roles of CNS-related genes in a predisposition to elite athletic traits. These findings are summarized in Table 2.

## 4. Ethical Issues

There is a need to carefully consider the use of genetic information in light of ethical issues. Recent advances in genetic technologies have made it possible to identify genetic factors that potentially contribute to a predisposition to elite athletic status. These advances have also provided genetic-based novel insights into athletic potential and vulnerability to injury of a particular athlete, which would be helpful to determine the most suitable sports for an individual, effective training program, methods to prevent and reduce the risk of injury, and effective recovery/rehabilitation protocols [4,5]. Conversely, the potential disadvantages of obtaining genetic information should also be considered. Participation in sporting events has the potential to enrich the life of the participant. However, the recognition of “unfavorable” or “undesirable” genotypes for a particular athlete could reduce motivation for training and enjoyment of the sport. Genetic factors are contributors, rather than determinants, of elite athletic traits [4]. Nonetheless, elite athletic status can be attained by a combination of continuous efforts of the athlete, social support, and various environmental factors.

The ethics of interventions that potentially contribute to attainment of higher EC should also be considered. Recent studies have argued that tDCS is a form of “brain-doping” in light of the potential to improve athletic performance [52,53,54]. In addition, there is currently no method to detect the use of tDCS [52]. These concerns highlight the need to determine whether tDCS meets the criteria of a doping substance or method, as stated in the World Anti-Doping Code of the World Anti-Doping Agency [54,57].

## 5. Conclusions

Recent advances in genetic research have identified novel genetic variants related to brain activity that potentially contribute to a predisposition to elite athletic traits, as inherent brain activity could be involved in inherent elite athletic status. Notably, several common CNS functions were identified between rodents and humans. These findings would corroborate the significance of CNS functions that predispose humans to perform high levels of exercise, although attention should be paid to species differences between rodents and humans.

Studies have highlighted the effectiveness of reducing central fatigue as an intervention strategy for improving exercise performance. Central fatigue is caused by a decrease in the ability to send neuronal signals and negatively affects exercise performance [58]. Since the serotonergic system is closely implicated in central fatigue and lethargy, 5-HT in the CNS is a potential target for intervention [58]. Consistently, nutritional studies have focused on the effects of branched-chain amino acids (BCAAs) or carbohydrates (CHOs) on reducing the levels of 5-HT [58]. Interestingly, BCAA supplementation did not provide performance benefit despite the suppression of 5-HT metabolism during exercise, probably due to the negative effects of ammonia accumulation in the brain, whereas CHO supplementation resulted in a decrease in 5-HT metabolism, consistent with a delay in fatigue [58]. These findings suggest the effectiveness of nutritional intervention to reduce 5-HT levels, thereby delaying central fatigue. The genetic factors and mechanisms regulating the expression of genes involved in the serotonergic system in response to BCAAs, CHOs, or BCAAs and CHOs are unknown. However, the responsiveness of those genes to BCAAs, CHOs, or BCAAs and CHOs would be a novel factor contributing to mental health and athletic performance. Inherently elevated levels of activation of pathways that prevent ammonia accumulation would also correlate with mental control and exercise performance in the case of BCAA supplementation.

A systematic review showed the ergogenic effects of caffeine supplementation in combat sports [59]. Caffeine modulates CNS function by binding to adenosine receptors A_1_ and A_2a_ and inhibiting the parasympathetic nervous system, which leads to increased alertness and mood enhancement [59]. Caffeine also increases glycolytic activity during combat [59]. These effects of caffeine have been reported to improve the performance of combat athletes [59]. Conversely, a study showed no significant effect of caffeine ingestion on punching performance in professional mixed martial arts athletes [60]. Although genetic analyses were not conducted in this study [60], genetic factors involved in caffeine metabolism or signaling pathways may significantly affect inter-individual differences in the response to caffeine, similar to that seen for *COMT* genotype-related DA doses in the CNS [50]. Consistently, caffeine exhibits different physiological effects dose-dependently, and good responders to caffeine exist [59]. In this respect, the optimal caffeine dose for a mixed martial arts athlete may compensate for the *COMT* genotype and confer improved performance, although functional relationships between the responsiveness to caffeine and *COMT* genotypes warrant further investigation.

In elderly individuals or those with mild cognitive impairment, exercise elevated BDNF levels and improved cognitive function [61,62]. As the *BDNF* genotype potentially predisposes acquisition of elite athletic traits [35,37] and insulin-like growth factor (IGF-1) mediates the response to exercise on BDNF and cognitive function [61], IGF-1 might be related to the inherent elite athletic status through the enhancement of motor skill acquisition. Interestingly, a study showed that CA repeat polymorphism of the *IGF-1* promoter was associated with the structure of motor skills in young athletes [63]. Future studies are needed to address the roles of the *IGF-1* genotypes in acquiring motor skills as well as growth hormones and their receptors, which elevate BDNF levels, as they might be novel CNS function-related contributors in predisposing toward elite athletic traits.

**Figure 1:**
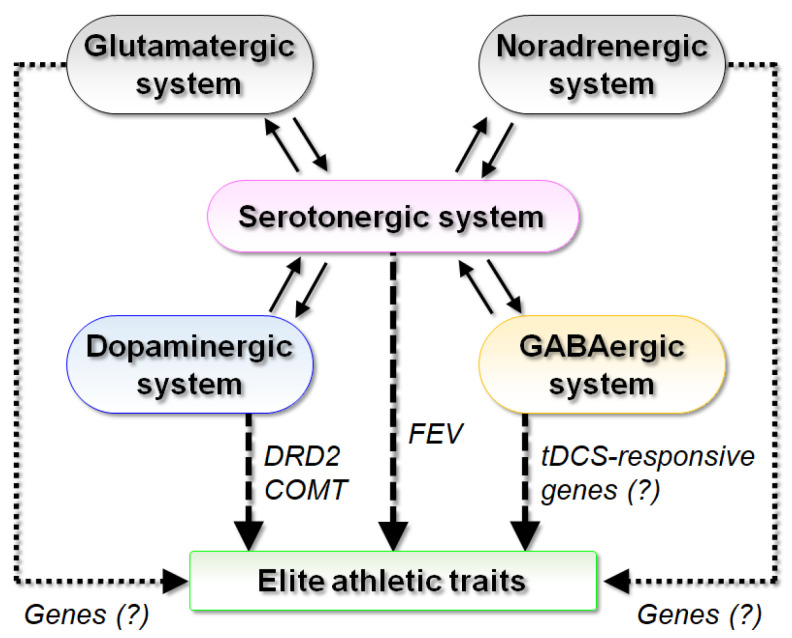
Potential implications of the CNS in a predisposition to elite athletic traits. The serotonergic system functionally interacts with multiple neurotransmitter systems, including the dopaminergic, GABAergic, glutamatergic, and noradrenergic systems. Genes related to these pathways (i.e., *FEV* in serotonergic system, *DRD2* and *COMT* in dopaminergic system, and potentially tDCS-responsive genes in GABAergic system) have potential roles in a predisposition to elite athletic status. Hence, further studies are warranted to identify genes associated with the glutamatergic and noradrenergic systems. *COMT*, catechol-O-methyltransferase; *DRD2*, dopamine D2 receptor; *FEV*, fifth Ewing variant; GABA, γ-aminobutyric acid; tDCS, transcranial direct current stimulation.

In light of recent findings regarding the relationship of neurotransmitters in the CNS and physical performance, DA, serotonin, and GABA are potential targets for modulation of neurotransmission in the CNS to improve athletic performance (Figure 1). In particular, the serotonergic system interacts with multiple neurotransmitter systems, including the dopaminergic, GABAergic, glutamatergic, and noradrenergic systems [64], to influence neuronal activities, such as modulating dopaminergic function, inhibiting or stimulating GABA release depending on the type of serotonin receptor, inhibiting glutamate release, and regulating the development of noradrenergic neurons and stimulating serotonin release by the noradrenergic system [64]. These interactions play significant roles in movement control by the basal ganglia and, thus, might be involved in the pathogenesis of Parkinson’s disease, a neurodegenerative disease characterized by resting tremors, rigidity, and bradykinesia [64]. In this respect, analysis of genes related to CNS dysfunction-related diseases characterized by derangement of motor and/or mental control could identify novel genotypes for a predisposition to elite athletic traits and vice versa. Future basic and clinical genetic studies targeting CNS function will reveal new avenues for the development of novel precision strategies to improve athletic performance, physical therapy regimens, and treatments of neurodegenerative diseases.

## Figures and Tables

**Table 1:** Functions of the central nervous system (CNS) potentially related to inherent exercise capacity (EC) in rats.

CNS Functions Potentially Related to EC	Genes Involved/Analyzed	Animals and Related EC	Reference
Pain analgesia system	Not identified	Rats with inherent high EC	[6]
Serotonergic system	Not identified	Rats with inherent high EC	[18]
Dopaminergic system	Not identified	Neuroplasticity in rats with inherent high EC	[19]
Thermoregulatory system	Not identified	Rats with inherent high EC	[20]

**Table 2:** CNS-related genes potentially involved in a predisposition to elite athletic traits in humans.

Genes/Genotypes	Potential CNS Functions Related to Athletic Traits	References
Brain-derived neurotropic factor (*BDNF*) A/G (rs6265)	Modulation of interhemispheric transfer of a procedural motor skill	[35,37]
Dopamine D2 receptor (*DRD2*) A/A (rs1076560)	Motor learning and performance	[35,38]
β 1 adrenergic receptor (*ADRB1*) C/T (rs776439595)	Sleep regulation	[39]
Fifth Ewing variant (*FEV*) G/G (rs860573)	Elevated serotonergic activity for optimal performance	[34]
Catechol-O-methyltransferase (*COMT*) G/G (rs4680)	Attainment of optimal dopamine dose under athletic competition	[50]

## Data Availability

Not applicable.

## Abbreviations

5-HIAA	5-hydroxyindoleacetic acid
5-HT	5-hydroxytryptamine
CNS	Central nervous system
CPu	Caudate putamen
DA	Dopamine
DOPAC	3,4-dihydroxyphenylacetic acid
EC	Exercise capacity
GABA	γ-aminobutyric acid
NAc	Nucleus accumbens
PFC	Prefrontal cortex
SNPs	Single nucleotide polymorphisms
tDCS	Transcranial direct current stimulation
